# Use of Milk

**Published:** 1854-01

**Authors:** 


### *Milk.*

Many persons imagine that the milk of cows is one of the most healthful of all articles, and yet it is a great mistake, except under certain limitations. By stout, strong, hardy, industrious out-door working men it may be used advantageously for breakfast and dinner, but, except in tea and coffee, and now and then half a glass for breakfast or dinner, it is not a proper article of food for invalids. In many instances patients have said to me, "I used to be a dear lover of milk, but I thought it made me bilious, and I have ceased using it altogether." This is the common-sense observation of ordinary men, one that, without any theory and against a life-time of prejudice, has forced itself upon the attention.

The rule that a man may eat almost anything with impunity, applies to one in good health, eating in moderation, according to the quality of the food, but when an invalid is to be fed, very different principles are to govern.

In all that I may say, I ask credence for nothing, except in proportion as it is followed up by the argument of *whole facts.*

